# SSPACE-LongRead: scaffolding bacterial draft genomes using long read sequence information

**DOI:** 10.1186/1471-2105-15-211

**Published:** 2014-06-20

**Authors:** Marten Boetzer, Walter Pirovano

**Affiliations:** 1 BaseClear B.V., Genome analysis and technology department, Einsteinweg 5, Leiden 2333 CC, The Netherlands

**Keywords:** De novo assembly, Scaffolding, Single molecule sequencing, Pacific biosciences, Genome finishing

## Abstract

**Background:**

The recent introduction of the Pacific Biosciences RS single molecule sequencing technology has opened new doors to scaffolding genome assemblies in a cost-effective manner. The long read sequence information is promised to enhance the quality of incomplete and inaccurate draft assemblies constructed from Next Generation Sequencing (NGS) data.

**Results:**

Here we propose a novel hybrid assembly methodology that aims to scaffold pre-assembled contigs in an iterative manner using PacBio RS long read information as a backbone. On a test set comprising six bacterial draft genomes, assembled using either a single Illumina MiSeq or Roche 454 library, we show that even a 50× coverage of uncorrected PacBio RS long reads is sufficient to drastically reduce the number of contigs. Comparisons to the AHA scaffolder indicate our strategy is better capable of producing (nearly) complete bacterial genomes.

**Conclusions:**

The current work describes our SSPACE-LongRead software which is designed to upgrade incomplete draft genomes using single molecule sequences. We conclude that the recent advances of the PacBio sequencing technology and chemistry, in combination with the limited computational resources required to run our program, allow to scaffold genomes in a fast and reliable manner.

## Background

Since the introduction of Next Generation Sequencing (NGS) much attention has been given to the development of *de novo* assembly software. The aim is to combine the short sequencing reads into a minimum number of linear stretches, though this goal is only partially achieved by draft assembly methods such as Velvet [1], SOAPdenovo [2] or ABySS [3]. Consequently more emphasis has been placed on so-called “genome finishing” tools which aim to reduce the number of contiguous sequences, for instance by the use of distance information between short paired reads. Indeed draft assemblies can be significantly enhanced when applying a scaffolding routine [4-6]. Nonetheless these protocols can still not overcome major hurdles such as (large) repeats and low-coverage regions. Alternatively the use of long read sequences as offered by the PacBio RS methodology can potentially solve complex genomic situations, yet the algorithmic implementation still suffers from a relatively high error rate. At present so-called Continuous Long Reads (CLR) may even exceed a size of 20 kbp at the cost of an error rate of approximately 15%, whereas the shorter Circular Consensus Reads (CCS) can span maximally 3 kbp though at a 2.5% error rate [7].

It is a common thought that the quality of the CLR reads is yet insufficient for high quality assemblies unless the genome coverage is very high. Recently, Chin et al. [8] have presented a novel non-hybrid method called HGAP. Here only CLR reads are used to finish bacterial genomes giving the advantage of a single library preparation. Although a sequence coverage of approximately 50× is sufficient to correct the error rate, a higher coverage is needed to span repeated elements. Also a significant manual intervention is needed to polish the genomes (*i.e.* manual error correction). From a cost perspective it means that a relatively large budget is needed to close a single genome using only CLR reads, especially when (larger) eukaryotes are studied.

Contemporarily it has been proposed to use a hybrid assembly approach in the attempt to enhance error-prone CLR reads. In principle this is possible using either PacBio CCS reads or short read NGS data (or a combination of both). To date a few algorithms have been released that are capable of upgrading PacBio CLR data with high accuracy data from CCS or short read NGS data, among which PacBioToCA [9] and LSC [10]. These are further incorporated into hybrid assembly methods such as Celera [11], MIRA [12] and ALLPATHS-LG [13]. Even though promising results have been obtained, the error-correction step with short reads requires a sufficient read length (>75 bp) and sequencing depth, as well as large computational demands. Notably the PacBioToCA (PBcR) correction pipeline also supports non-hybrid PacBio assemblies in case C2 or newer sequence reads are used.

As regards scaffolding, the AHA (A Hybrid Assembler) strategy is currently the most widely used approach: the method employs the usage of the CLR reads only for scaffolding of pre-assembled contigs [14], yet it generally results in incomplete assemblies and is not designed for large-scale genome assembly applications given the long runtime and input restrictions (the current draft genome size limit for AHA in the SMRT Analysis suite v2.0 is 160 Mb and 20,000 contigs). Recently a novel hybrid assembly tool has been released, Cerulean [15], which uses ABySS [3] contig graph information and uncorrected CLR reads to create genome scaffolds. Despite of the promising results obtained, the requirement to use ABySS for the draft assembly is a limiting factor as other methods might generate a better draft [16]. Finally dedicated tools have been developed to close gaps within scaffolds using PacBio reads, among which PBJelly [17]. A future release of this software aims to also incorporate a scaffolding module. Given the limitations encountered for short read sequencing (in terms of length) and PacBio long read sequencing (in terms of quality), the complete closure of prokaryotic and eukaryotic genomes is still a relatively expensive and difficult task. Indeed it can be observed that most genome submissions consist of hundreds (or thousands) of unconnected contigs.

Here we present a hybrid approach to scaffold draft genomes in a cheap, fast and reliable manner. In brief the algorithm consists of three steps: 1) Alignment of long reads against the pre-assembled contigs (or scaffolds) 2) Computation of contig linkage from the alignment order and 3) Scaffolding of contigs (or scaffolds) including placement of repeated elements. On a number of test datasets we show our method, called SSPACE-LongRead, outperforms the AHA method in terms of genome completeness. Importantly the input draft assemblies were constructed using only a single Illumina MiSeq or Roche 454 library and a complementary PacBio RS C2 long read library. The combination of barcoding possibilities on the PacBio RS instrument and the relatively low coverage needed by the SSPACE-LongRead algorithm opens novel ways of scaffolding genomes at reduced costs compared for instance to the use of NGS mate paired-end libraries. Our software is setup in a user-friendly manner and is suited for analysis on small computing systems given the very fast runtime. Academics can request a free copy at http://www.baseclear.com/bioinformatics-tools/.

## Results and discussion

In order to test the performance of SSPACE-LongRead and compare it to PacBio’s AHA scaffolds, we have performed in-depth analysis on six bacterial datasets. These include *B. trehalosi*, two *E. coli* strains (K12-MG1655 and O157:H7), *F. tularensis*, *B. trehalosi, M. haemolytica* and *S. enterica*. Details are described in Table 1 and the Methods section. For each organism paired-end Illumina MiSeq reads (on average 90-100× coverage) were used to create two draft assemblies: one with Ray [18] and one with CLCbio *de novo* assembly software (CLC bio, Aarhus, Denmark). An alternative draft assembly was made with Newbler (Roche) using only Roche 454 reads (on average 45-50× coverage), except for *M. haemolytica* for which no 454 reads were available. Subsequently the draft contigs were scaffolded using uncorrected PacBio RS long CLR reads (up to 200× coverage) with both SSPACE-LongRead and AHA. The results are displayed in Table 2.

**Table 1:** Statistics of the sequence datasets used for the comparative study

	**Illumina MiSeq reads**			**Roche 454 reads**			**PacBio RS reads**		
**Organism**	**Total reads (Kbp)**	**Total bases (Mbp)**	**Mean read length (bp)**	**Total reads (Kbp)**	**Total bases (Mbp)**	**Mean read length (bp)**	**Total reads (Kbp)**	**Total bases (Mbp)**	**Mean read length (bp)**
*E. coli* K12 MG1655	3,046.4	460.0	151	44.94	232.0	516	383.5	929.1	2,422
*E. coli* O157:H7	3,794.9	548.5	144	946.2	219.1	231	403.9	1,100.3	2,724
*B. trehalosi*	1,718.2	249.2	145	351.9	190.7	542	205.1	499.9	2,437
*M. haemolytica*	1,724.4	249.4	144	NA	NA	NA	176.0	531.2	3,019
*F. tularensis*	926.7	199.2	214	178.9	74.5	416	176.4	399.8	2,266
*S. enterica*	1,943.8	279.8	143	351.5	127.1	361	394.7	1,000.2	2,534

Overall a 90-100× Illumina MiSeq and 45-50× Roche 454 genome coverage is used. For the PacBio dataset an input coverage of ~200× is available.

**Table 2:** Genome reconstruction of 6 bacterial genomes using different sequencing platforms and assembly strategies

**Organism**	**Assembler**	**Scaffolder**	**Expected scaffolds**	**Final scaffolds**	**Unaligned scaffolds**	**Sum (bp)**	**N50**	**Gap size (bp)**	**Indels**	**Rearran-gements**	**Runtime**
***B. trehalosi***	Ray	-	Unknown	34	-	2,384,099	212,852	0	-	-	-
		AHA	Unknown	21	-	2,390,466	245,559	6,367	-	-	110 min
		SSPACE LongRead	Unknown	7	-	2,410,351	1,215,562	8,899	-	-	16 min
	CLC	-	Unknown	62	-	2,361,409	146,347	0	-	-	-
		AHA	Unknown	36	-	2,389,684	222,352	16,915	-	-	118 min
		SSPACE LongRead	Unknown	6	-	2,395,822	1,361,277	8,650	-	-	19 min
	***Newbler***	-	Unknown	58	-	2,362,898	117,742	0	-	-	-
		AHA	Unknown	21	-	2,391,876	505,738	12,781	-	-	117 min
		***SSPACE LongRead***	***Unknown***	***5***	***-***	***2,393,982***	***1,317,689***	***7,692***	***-***	***-***	***16 min***
***E. coli K12***	Ray	-	1	99	0	4,583,740	95,924	0	0	2	-
		AHA	1	57	0	4,632,207	220,952	32,147	2	2	194 min
		SSPACE LongRead	1	11	0	4,636,946	570,605	30,741	1	9	28 min
	***CLC***	-	1	126	0	4,554,695	88,183	0	-	-	-
		AHA	1	57	0	4,636,666	497,336	34,587	2	6	214 min
		***SSPACE LongRead***	***1***	***1***	***0***	***4,642,513***	***4,642,513***	***18,788***	***3***	***8***	***28 min***
	Newbler	-	1	80	0	4,567,139	117,490	0	-	-	-
		AHA	1	12	0	4,652,318	3,320,126	45,090	6	14	201 min
		SSPACE LongRead	1	2	0	4,635,316	3,716,545	7,793	7	10	32 min
***E .coli O157:H7***	Ray	-	10	144	1	5,432,073	112,112	0	-	-	-
		AHA	10	110	1	5,475,255	227,802	34,035	1	2	226 min
		SSPACE LongRead	10	38	1	5,845,919	348,040	58,068	2	23	31 min
	***CLC***	-	10	293	13	5,335,444	105,156	0	-	-	-
		AHA	10	238	8	5,437,860	201,528	42,214	4	9	312 min
		***SSPACE LongRead***	***10***	***33***	***2***	***5,539,369***	***1,172,184***	***51,676***	***13***	***17***	***32 min***
	Newbler	-	10	279	14	5,322,767	142,438	0	-	-	-
		AHA	10	209	8	5,471,954	254,465	65,936	5	9	297 min
		SSPACE LongRead	10	39	3	5,565,065	703,452	75,126	11	34	37 min
***F. tularensis***	Ray	-	3	100	0	1,806,660	25,623	0	-	-	-
		AHA	3	38	0	1,859,591	82,151	47,651	1	5	95 min
		SSPACE LongRead	3	8	0	1,886,509	279,967	27,386	1	8	14 min
	CLC	-	3	110	1	1,780,141	25,117	0	-	-	-
		AHA	3	53	1	1,844,586	63,063	50,494	0	6	104 min
		SSPACE LongRead	3	7	1	1,877,533	444,696	19,639	2	6	18 min
	***Newbler***	-	3	316	0	1,653,291	8,912	0	-	-	-
		AHA	3	61	0	1,965,997	69,167	255,189	7	7	95 min
		***SSPACE LongRead***	***3***	***7***	***0***	***1,867,474***	***480,062***	***160,504***	***16***	***13***	***14 min***
***M. haemolytica***	Ray	-	Unknown	80	-	2,639,260	75,015	0	-	-	-
		AHA	Unknown	44	-	2,676,952	108,006	25,336	-	-	148 min
		SSPACE LongRead	Unknown	14	-	2,682,588	703,034	29,889	-	-	21 min
	***CLC***	-	Unknown	129	-	2,630,768	63,442	0	-	-	-
		AHA	Unknown	41	-	2,769,108	239,432	73,082	-	-	166 min
		***SSPACE LongRead***	***Unknown***	***8***	***-***	***2,742,871***	***1,996,208***	***33,032***	***-***	***-***	***25 min***
***S. enterica***	Ray	-	4	119	2	4,972,739	90,542	0	-	-	-
		AHA	4	40	2	5,012,323	203,631	34,496	0	4	190 min
		SSPACE LongRead	4	20	2	5,112,337	488,483	27,988	0	6	28 min
	CLC	-	4	238	5	4,974,534	43,328	0	-	-	-
		AHA	4	62	4	5,064,555	376,354	68,292	3	7	200 min
		SSPACE LongRead	4	7	3	5,038,082	3,235,544	21,588	6	2	34 min
	***Newbler***	-	4	101	12	4,990,994	372,513	0	-	-	-
		AHA	4	69	12	5,040,830	787,589	30,907	2	6	193 min
		***SSPACE LongRead***	***4***	***4***	***12***	***5,036,244***	***3,729,047***	***10,430***	***3***	***11***	***29 min***

In italic-bold the platform/strategy that leads to the lowest amount of assembled scaffolds is highlighted. The number of expected scaffolds refers to the number of chromosomes plus the number of plasmids present in the reference genome (if available). Generally the combination 1) draft assembly using CLCbio for Illumina MiSeq reads or Newbler for Roche 454 reads and 2) scaffolding using SSPACE-LongRead for PacBio CLR reads gives the best results in terms of closure and time. Notably some draft assembly contigs are not covered with PacBio reads (such as PhiX control or bacterial host sequences). The number of errors introduced during scaffolding is only limited and often are a consequence of true variations between the sequenced library and the earlier deposited reference genome.

### SSPACE-LongRead effectively reconstructs (nearly) complete genomes

The assembly statistics show that both AHA and SSPACE-LongRead are able to significantly reduce the amount of input draft contigs using error-prone PacBio RS CLR reads as guidance. Overall the total assembly length remains relatively stable. It is apparent that SSPACE-LongRead is better able to reconstruct continuous genome segments given the final number of scaffolds is generally lower than 10. In practice this generally means a reduction of the initial amount of contigs by at least 90%. It should also be remarked that the runtime of these tools differs by a factor of 7, making our software more suited for scaffolding genomes on smaller computing systems. In terms of accuracy (through comparison with the corresponding reference genomes, not available for *B. trehalosi* and *M. haemolytica*) our software introduces some more errors compared to AHA but this is clearly explained by the more conservative approach of AHA (leading to less contig connections). Also it should be remarked that some of the apparent errors are actually true variations between the sequenced strain and the reference genome, an issue which is also explained by Koren et al. [19] as for each sequenced strain a close (but not necessarily the exact) reference was selected for quality assessment.

Not all input contigs were covered by PacBio reads (*i.e.* no significant alignment could be found between the draft assembly and the PacBio reads). This may be explained by the possible presence of short sequences such as plasmids which can not be captured by the long insert PacBio libraries. For this reason a hybrid assembly approach provides more complete assemblies than experiments that include only one PacBio library (as a compromise needs to be made between a large insert library – generating larger reads and better genome closure – and a short insert library to capture also short plasmids). Another explanation can be given by presence of different DNA sources in the NGS library. For instance, in the case of *S. enterica* a relatively high number of sequences is generated after scaffolding the Newbler draft assembly. However 12 of these input contigs are not covered by PacBio reads and surprisingly most of these show a high similarity with *B. Taurus* after a BLAST [20] analysis on the NCBI nr database. Other contigs uncovered by PacBio show similarity with the 5386 bp genome of phi X 174 (phiX), which is used by Illumina as a control to validate the quality of a run. In principle the corresponding reads should be removed from the MiSeq runs prior to downstream analysis.

### Genomes become less fragmented also at a low PacBio RS CLR read coverage

**Figure 1:**
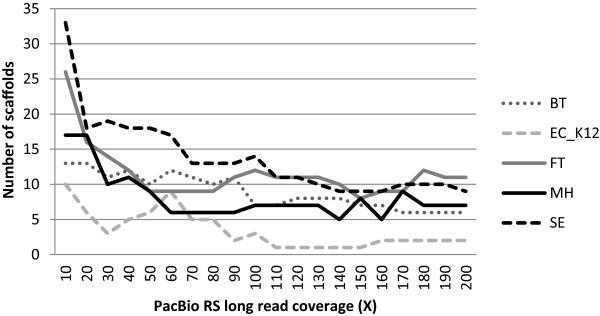
**The effect of PacBio RS long read coverage on genome closure.** Results are displayed for SSPACE-LongRead based on the CLCbio draft assembly for 5 organisms. For all samples the addition of PacBio reads has a positive effect and leads to a significant contig reduction. In general a 50× coverage is sufficient to scaffold over most gaps, though ideally a 110-160× coverage is required to guarantee an optimal performance of our software. Arguably a higher coverage (>160×) leads to more fragmented genomes, which is likely due to the increased complexity of the assembly graph.

In Figure 1 it can be observed that a higher PacBio RS long read coverage leads to less fragmented genomes. Nonetheless a coverage of 50× is generally sufficient to reduce the number of genome fragments to less than 10 scaffolds. The best assembly results are yielded at a coverage value between 110-160×. A higher coverage does not seem to improve the outcomes, instead this leads to more fragmented genomes. From a cost-perspective, the PacBio XL-C2 chemistry specifications (about 300 Mbases per SMRT cell) imply that for a small bacteria genome closure can be achieved using one SMRT cell in combination with *e.g.* one MiSeq paired-end library. More recent improvements of the sequencing chemistry (P4-C2 and P5-C3) aim to further enhance the data throughput (>500 Mbases) and the read length (>20 kbp). Nonetheless still a relatively high coverage is needed to completely finish a bacterial genome which is partly explained by the high error-rate which needs to be corrected for. Generally the alignment of PacBio RS long reads to a set of contigs yields low alignment scores and a non-conservative approach (based only a few reads that span consecutive contigs) will likely lead to misassemblies. Moreover it should be considered that after sequencing the number of long reads (e.g. more than 5 or 10 kbp), which are essential to overcome large repeats, is relatively small compared to the number of short reads (the median read length is currently between 2 and 5 kbp). Thus the contribution of long reads to the overall sequencing coverage is only limited. It is to be expected though that the total data yield per SMRT cell, as well as the mean read length, will significantly increase in the near future. Thus costs can be further reduced if samples are barcoded and sequenced together on the same SMRT cell. At this point a hybrid approach consisting of one short read paired-end and one long read PacBio library may eventually be the method of choice in terms of accuracy and costs compared to an alternative approach involving mate paired-end libraries. Last but not least, the computational time involved to close bacterial genomes allows high-throughput assemblies even on small compute systems. It should be underscored that the current study was performed using uncorrected PacBio RS CLR reads, thus bypassing a time-consuming error-correction step with short reads.

### Both the quality of the draft assembly and the selection of the assembly strategy are crucial for the success of an experiment

From Table 2 it can be observed that the assembly results are also largely dependent on the strategy chosen for constructing the draft assembly. Common criteria used to judge a draft assembly are the number of contigs (should be low) and the N50 value (should be high to indicate that at least 50% of the assembly is in contigs of at least this length/value). Nonetheless these quantitative measures do not necessarily guarantee the choice of the best assembly.It is therefore adviced to assess the quality of an assembly using a dedicated tool, such as QUAST [21], which gives a complete overview of different quality metrics. Yet also other factors can play an important role in the selection of the most appropriate input assembly. For example, assemblies constructed with Ray do show a higher N50 value and a lower number of contigs compared to those constructed with CLCbio or Newbler. Therefore it might be intuitive to choose the Ray contigs as input for SSPACE-LongRead scaffolding. Nonetheless from the SSPACE-LongRead statistics it appears to be easier to reconstruct complete genomes from CLCbio or Newbler contigs. From further investigations (data not shown) we noticed that assemblies constructed with Ray contain repeated segments which are especially present at the contig edges. Similar observations were made for draft assemblies constructed from Illumina MiSeq reads with SPAdes [22] and MaSuRCA [23] (data not shown): the amount of contigs was lower or similar to that observed for CLCbio and Newbler, though the errors in the draft assembly led to more (erroneous) scaffolds. Although SSPACE-LongRead performs well on all tested draft assembly strategies, the user should preferably choose an assembly method that splits contigs at repeat boundaries. Consequently the assessment of the most appropriate draft assembly should not be solely based on the assembly quality-metrics, as the extent of the genome fragment reduction also depends on the draft assembly strategy.

## Conclusions

We propose a novel tool for scaffolding pre-assembled contigs using long read information. We show that even error-prone PacBio RS CLR reads can be well used to connect contigs, place repeats and consequently reconstruct bacterial genomes in less than 10 segments when using SSPACE-LongRead. Importantly only two libraries are needed for the hybrid assembly of a bacterial genome. One Illumina MiSeq or Roche-454 paired-end library is sufficient for the construction of a proper draft assembly using a state-of-the-art De Bruijn-graph or Overlap-consensus layout assembler, although the final quality is influenced by the exact method chosen. Importantly the number of contigs (or other metrics such as the N50 value) may not be the best criteria for evaluating the best draft assembly. Indeed we show that, although draft assemblies created with Ray yield fewer contigs, draft assemblies created with CLCbio or Newbler software lead to more closed genomes after scaffolding. It can be therefore argued that ideally the user should choose a draft assembly method that places repeated elements into separate contigs: whereas CLCbio and Newbler tend to automatically break reads and contigs at repeat boundaries, Ray and SPAdes tend to merge unique and repeated sequences together in the same contigs.

We argue that one PacBio RS library is sufficient to nearly finish the bacterial assembly. In this paper we show promising results using a PacBio coverage of 50×, a CLR error-rate of 15% and a mean read length between 2-3 kb. It is likely that further improvements of the sequencing platforms and chemistry will show additional improvements, *i.e.* a complete genome closure at lower costs. To this regard also the introduction of a barcoding system for PacBio libraries will help to use SMRT cells more efficiently and enlarge the capacity per sequencing run. Also our method can be used for possible new sequencing platforms, such as the Illumina Moleculo and Oxford Nanopore systems, given that the input format (FASTA or FASTQ) is standardized and not specifically bound to a platform.

Contemporarily it can be foreseen that additional methods will appear that perform assemblies using only long read information. At present the HGAP assembly method seems to be the only such available strategy, however there are some major issues with the overlap-consensus implementation (which requires an accurate prior estimation of the expected genome size) and the introduction of erroneous duplications (which need to be manually removed [24]). Moreover our SSPACE-LongRead method is optimized for high-throughput experiments (given the simple running mode of the script and short runtime) and can be the method of choice for upgrading existing draft genomes in a cost-effective manner. Also we expect benefits for (larger) eukaryotic organisms for which genome submissions generally consist of highly fragmented chromosomes.

We feel the current study opens new ways to address the genome assembly question and can positively contribute to the reconstruction of more complete genomic contexts.

### Availability and requirements

•**Project name:** SSPACE-LongRead

•**Project home page:**http://www.baseclear.com/bioinformatics-tools/

•Operating systems: All major Linux platforms

•**Programming languages:** Perl, C++ (the latter is required for BLASR, see below)

•**Other requirements:** BLASR for the alignment of long reads [22]

•**License:** BaseTools software license

•**Any restrictions to use by non-academics:** commercial licence needed

## Methods

**Figure 2:**
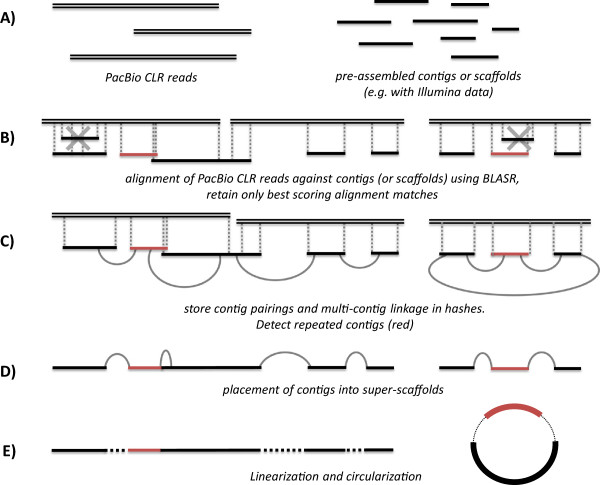
**Overview of the SSPACE-LongRead scaffolding algorithm. A)** The input consists of a set of pre-assembled contigs (or scaffolds) in FASTA format and a set of PacBio CLR reads (in FASTA or FASTQ format). **B)** The PacBio CLR reads are aligned against the contigs using BLASR and only the best alignment matches are kept. In red a repeated element is indicated. **C)** Contig pairings and multi-contig linkage information is stored, from this information also repeated elements are detected. **D)** Based on the pairing and linkage information, contigs are ordered, oriented and connected into scaffolds. **E)** A post-processing step performs the final linearization and circularization.

The SSPACE-LongRead methodology can be summarized in a few steps which are described below and summarized in Figure 2. The pseudocode is summarized in Additional file 1: Figure S1.

### Software input

The user needs to create a draft assembly using a *de novo* assembly method of choice (*e.g.* Velvet [1], SOAPdenovo [2], Ray [18], CLCbio (CLC bio, Aarhus, Denmark) or Newbler (Roche)). Optionally the user may also provide scaffold sequences generated with dedicated software (*e.g.* SSPACE [5] or SOPRA [4]). The resulting contigs or scaffolds (in FASTA format) are to be provided as input to SSPACE-LongRead software together with a set of long reads in FASTQ or FASTA format (*e.g.* PacBio CLR reads). Note that in our study we observe that SSPACE-LongRead obtains the best results if the draft assembly is constructed with CLCbio or Newbler as these tend to better split contigs at repeat boundaries (see the Results and Discussion section for additional explanations).

### Alignment of long reads against the pre-assembled contigs (or scaffolds)

Each long read is aligned against the pre-assembled contigs with BLASR [25] resulting in a local alignment and corresponding similarity score. For gap-estimation purposes, the local alignments are extended to generate a full contig match. In order to remove false-positive alignments, contigs that display a (partial) overlap with a contig that has obtained a higher alignment score are iteratively removed from the dataset (Figure 2, step B). The minimum overlap (in bp) required to remove a contig from the alignment is defined by parameter *-g* (default value = 200).

### Computation of contig linkage from the alignment order

The remaining contigs are sorted based on their alignment position on the long reads. Subsequently the contig distance and orientation is computed. Each contig-pairing and multi-contig linkage is stored in a hash: the preferred pairings are retained by removing contig-links which are also found within a multi-contig path of another contig-link. For example, if there are two paths, A- > B- > C- > D and A- > C- > D, the linkage of A- > C is removed. The ambiguous paths are mainly explained by the fact that the BLASR alignment tool generally can not resolve (align) low-quality alignment regions of PacBio reads. If multiple paths remain, a ratio is calculated between the two best alternatives. Ambiguous pairings are solved using the multi-contig linkage information, the unsolved pairings are flagged as repeated elements.

### Scaffolding contigs into scaffolds

The contigs are now connected into linear stretches where repeated elements are placed based on the multi-contig linkage information. Repeated elements on the edges of the linear stretches are removed. As a post-processing step, the non-repeated edges of the preliminary scaffolds are reused to find connections between other preliminary scaffolds. Finally the gap-size between each contig is calculated: if this value is negative, and an overlap is found, contigs are merged; if this value is positive, a gap is inserted between contigs (the gap is represented by one or more undefined ‘N’ nucleotides depending on the gap-size).

### Software output

The final linear assembly is represented in an easily interpretable FASTA file. In addition a AGP (Accessioned Golden Path) file is generated which describes the contig order within the scaffolds. The latter files can be readily used for NCBI genome submissions. Also a summary file containing statistics of the assembly process and final assembly structure is provided in TEXT format.

### Datasets

In total six bacterial datasets were used for testing the performance of the software. These comprise Illumina MiSeq, Roche-454 and PacBio RS reads from *Escherichia coli* (K12 MG1655), *Escherichia coli* (O157:H7 F8092B), *Bibersteina trehalosi* (USDA-ARS-USMARC-192), *Mannheimia haemolytica* (USDA-ARS-USMARC-2286), *Francisella tularensis* (99A-2628) and *Salmonella enterica* (Newport SN31241). Datasets are downloaded from http://www.cbcb.umd.edu/software/PBcR/closure/index.html and further described in Koren et al. (2013). Dataset statistics are displayed in Table 1. To assess the assembly correctness we used close reference genomes deposited in the NCBI database (*E. coli* K12 MG1655 = NC_000913, *E. coli* O157:H7 = NC_002127, NC_002128, NC_002695, *F. tularensis* = NC_008369, *S. enterica* = NC_011079, NC_011080, NC_009140). For *B. trehalosi* and *M. haemolytica* no reference genome is currently available.

### Assembly procedure

Draft assemblies of Illumina MiSeq data were constructed using Ray version 2.3.0 [18] and the CLCbio *de novo* assembler version 6.5.1 (CLC bio, Aarhus, Denmark) using for each program a *k-mer* setting of 31. Draft assemblies of Roche-454 data were constructed using Newbler version 2.8 (Roche) as described in Koren et al. [19]. Scaffolding was performed using AHA [14] which is part of the SMRT Analysis Package version 2.0 and SSPACE-LongRead. For the latter software we required a minimal estimated overlap of 200 bp (option *-g*) between the contigs in order to avoid false positive alignments. For Ray the minimal estimated overlap was set to 500 bp since Ray tends to include repeated elements on contigs edges: as a result a larger overlap between the contigs is observed.

### System

All analysis were performed on a 48 Gb Linux machine (Intel Xeon X5650, 2.67 GHz).

### Sourcecode

SSPACE-LongRead is written in Perl and runs on all major Linux platforms. A pseudocode of the algorithm is given in Additional file 1: Figure S1.

## Competing interests

The authors declared that they have no competing interests.

## Authors’ contribution

MB and WP authors contributed to the design of the study and the interpretation of the results. MB performed the implementation, WP wrote the manuscript. Both authors read and approved the final manuscript.

## Supplementary Material

Additional file 1 **Pseudocode SSPACE-LongRead.** In this figure an overview is given of the operating principle of the SSPACE-LongRead algorithm. Click here for file
