# Supplementary material for: SSPACE-LongRead: scaffolding bacterial draft genomes using long read sequence information
Source: BMC Bioinformatics. 2014 Jun 20;15:211. doi: 10.1186/1471-2105-15-211 (PMC4076250; doi:10.1186/1471-2105-15-211)
Supplement: Additional file 1 — Pseudocode SSPACE-LongRead. In this figure an overview is given of the operating principle of the SSPACE-LongRead algorithm. [file 1471-2105-15-211-S1.pdf]

1) Align long reads against the pre-assembled contigs (or scaffolds)
   # Align each long read against the pre-assembled contigs with BLASR
   # Extend each local alignment to full contig size
   # Iteratively remove contigs with (partial) overlap to contig with higher alignment score

2) Compute contig linkage from alignment order
   # Sort contig order based on alignment positions on long reads
   # Calculate the inter-contig distance and orientation
   # Store contig-pairing and multi-contig linkage
   # Retain preferred pairings based on majority voting
   # Solve ambiguous pairings using multi-contig linkage information
   # Flag remaining ambiguous pairings as repeats

3) Scaffold contigs
   # Connect linear contig links
   # Place repeated elements based on multi-contig linkage information
   # Attempt to further connect linear links using multi-contig linkage information
   # Finished genome
   # Calculate gap-size and eventually merge contigs (if negative gap-size)
   # Search for possible circularization
